# O096: Care of patients with tracheostomy

**DOI:** 10.1186/2047-2994-2-S1-O96

**Published:** 2013-06-20

**Authors:** M Abraham

**Affiliations:** 1 Nursing, Infection Control Nurse, Apollo Hospitals, Hyderabad, India

## Background

Tracheostomy is common in the acute care facility today. The care for these patients is challenging for the nurses in the ward where knowledge and skill is tested and often lacking resulting in emergency shift of these patients to ICU. As we found it difficult to care for this group in the wards inspite of continous training, we decided to have a dedicated tracheostomy ward for patient safety and better outcome. As a part of training, this module on tracheostomy care was developed by the medical ICU nurses.

## Introduction

Tracheostomy is common in the acute care facility today. The care for these patients is challenging for the nurses in the ward where knowledge and skill is tested and often lacking resulting in emergency shift of these patients to ICU. Often chronic patients tend to be overlooked in Infection control practices (ICP)aspect. This leads increased Health care associated infections (HAI) along with increased morbidity & mortality.

## Objectives

To reduce the HAI in tracheostomy patients by ensuring proper infection control practices implementation by effective training module.

## Methods

As we found it difficult to care for this group in the wards inspite of continous training, we decided to have a dedicated tracheostomy ward for patient safety and better outcome. As a part of training, a module on tracheostomy care was developed by the medical ICU(MICU) nurses. Infection control issues are discussed in these patients as they are patients on long term care All the sisters who take care of patients having Tracheostomy are educated with the help of this audio visual medium. Also hands on training is provided with the help of a mannequin.

## Results

The care is improving in these patients. This is reflected in lower HAI rates and decrease in number of patients returning to the ICU’s from wards with tube blocks and aspirations.

## Conclusion

Continuous training is the most important part for sustaining ICP in a healthcare setup. We identified the tracheostomy patients as those falling in the high risk category for HAI. We could successfully train our sisters and implement the tracheostomy care practices and bring down the HAI rates.

## Disclosure of interest

None declared.

